# Adrenal lesions in patients with abdominal multitrauma

**DOI:** 10.1007/s12020-025-04321-9

**Published:** 2025-06-21

**Authors:** Anna Kistner, Lisa Kekonius, Jan Calissendorff, Seppo Koskinen, Henrik Falhammar

**Affiliations:** 1 Department of Molecular Medicine and Surgery, Karolinska Institute, Stockholm, Sweden; 2 Department of Nuclear Medicine and Medical Physics,, Karolinska University Hospital, Stockholm, Sweden; 3 Department of Endocrinology, Karolinska University Hospital, Stockholm, Sweden; 4 Department of Clinical Science, Intervention, and Technology, Division of Radiology, Karolinska Institute, Stockholm, Sweden; 5 Department of Radiology, Karolinska University Hospital, Stockholm, Sweden

**Keywords:** Adrenal incidentalomas, adrenal tumor, adrenal hematomas, CT imaging, follow-up, survival

## Abstract

**Purpose:**

Most adrenal lesions are less than 2 cm and can easily be ignored during primary review of computed tomography (CT)-scans with another question. Furthermore, adrenal lesions, including adrenal hematomas, can easily be missed in patients with multitrauma. We aimed to determine the incidence of adrenal lesions in patients with multitrauma.

**Methods:**

This retrospective, single-center cohort analysis included 1373 patients with multitrauma (71.2% males) with a mean age of 42 (95%CI 41–43) years. One radiologist re-examined all multitrauma CT performed 2013–2017 using thin sliced imaging in at least two depictions. A second radiologist re-examined patients where an abnormality was found. Adrenal abnormalities were labelled adrenal lesions (size ≥10 mm) or hematomas, respectively. Clinical data were collected.

**Results:**

The adrenal lesion prevalence was 2.7% (37/1373, 78% males, 22% bilateral). Of these 27% (10/37) were mentioned in the radiological report, and in those with a size ≥15 mm (n = 13), 38% (5/13) were mentioned. Of the unilateral adrenal lesions 86% (25/29) were left-sided. In the adrenal lesion group during the mean follow-up time of 75 (61–90) months none had surgery, 6% had hormones measured (all normal), and 7% had repeated adrenal imaging. The adrenal hematoma prevalence was 5.0% (68/1373, 10% bilateral). No patient had both an adrenal hematoma and an adrenal lesion. Of the unilateral hematomas 42% (26/62) were not mentioned. Of the hematomas 77% were right-sided. No patient with adrenal abnormalities had signs of adrenal insufficiency. 30-day survival rates in the adrenal lesion and hematomas groups were similar, but the hematoma group had higher injury severity score, but no longer length of hospital stay.

**Conclusions:**

The prevalence of adrenal tumors was 2.7% and adrenal hematomas 5.0%. Most lesions were not mentioned in the image report. Few had hormonal evaluation or adrenal imaging follow-up. Survival rates were similar between the two groups.

## Introduction

With the increasing use of computed tomography (CT) an increased number of incidental findings in the adrenals are detected. An incidental finding in the adrenals larger than or equal to 10 mm is called an adrenal tumor. An adrenal tumor found on imaging performed for other reasons than suspected adrenal disease is called adrenal incidentaloma [1]. A nonfunctional adrenal lesion is considered the most common adrenal tumor [2, 3]. Around 85% of all adrenal lesions will not be overtly functional [4], but a large proportion may exhibit mild autonomous cortisol secretion (MACS) [5]. Most adrenal lesions are small, below 2 cm, and can easily be ignored during primary review of CT-scans with another question. Studies of adrenal lesions have often been performed in middle-aged to older individuals [2]. Adrenal lesions are common in older patients >75 years (5–7%) [4]. In autopsy series the mean prevalence is 3% but may be a lot higher in some series [2]. In trauma series with many younger individuals the prevalence should be lower, reflecting the prevalence in the entire adult population. Few studies have investigated the prevalence of adrenal lesions in a middle-aged cohort. In a large screening study of patients participating in health checks in China with a mean age of 48 years a prevalence of barely 1.4% was found [6].

The size of the adrenal glands and its anatomical location in the stomach with surrounding adipose tissue makes them relatively protected from trauma. The acute adrenal trauma with bleeding is often oval and can be separated from an accidental adrenal adenoma by its attenuation. The acute bleeding most often leads to a higher attenuation compared with the adrenal adenoma (>40 Hounsfield unit, HU) [7].

Investigating adrenal lesions with unenhanced CT is a well-established way to characterize benign adrenal lesions. Unenhanced CT is recommended as a golden standard to estimate attenuation and to differentiate between benign adenomas and non-adenomas, with the combination of size (up to 40 mm) and attenuation (<10 HU) [8].

We hypothesize that it is relatively unusual with adrenal lesions accidently found in a multitrauma cohort, since most of the affected patients are young and healthy before the trauma. Our first aim was to investigate the prevalence of adrenal lesions ≧10 mm in our cohort. Our second aim was identifying the prevalence of adrenal hematomas. Our third aim was to make an assessment of our own work, and how many of the adrenal tumors or hematomas that were missed during primary and final review. Finally, we wanted to follow-up the patients with adrenal lesions to investigate the outcomes.

## Methods

### Study design

#### Participant selection and enrollment

This is a retrospective, single-center longitudinal cohort study. All patients with severe abdominal trauma during five years (2013–2017) were retrieved. All patients, >15 years with a trauma CT were included. The cases (n = 1373) were evaluated by two radiologists at the time of the trauma. Images were reviewed and re-evaluated particularly concerning the adrenal glands by an experienced radiologist (LK), and in cases with an adrenal abnormality these subjects were evaluated by a second experienced radiologist (AK), respectively. In eight cases where consensus could not be reached, a third experienced radiologist (SK) reviewed the imaging and made the final decision. The electronic medical records of all patients with an adrenal lesion were reviewed in detail, regarding adrenalectomy, apparent adrenal insufficiency, hormone evaluation (plasma cortisol, metanephrines, aldosterone and renin concentrations) and new imaging focusing on the adrenals.

Data from the participants reflecting age, sex, length of hospital stay (LOS), injury severity scale (ISS) and new injury severity scale (NISS) were collected. To calculate ISS the body is divided into six regions head and neck, face, chest, abdomen, extremities, and external. Each injury is assigned a score based on the sc abbreviated injury scale (from 1 (minor) to 6 (maximum injuries) and only the highest score in each region is counted. For ISS the three most severely injured ISS body regions are identified. The highest AIS severity code in each of these three regions is taken. Each of these AIS code is squared and the three squared numbers added. The New Injury Severity Score (NISS) is similar to the ISS, except that it is calculated using the patient’s three most severe injuries regardless of the body region in which they occur. For example, a patient with multiple gunshot wounds to the abdomen has injuries to their small intestine, liver and kidney, with an AIS severity codes of 3, 4 and 5, respectively. Since these three injuries are all to one body region (the abdomen), the ISS calculation would only include the kidney injury. However, the NISS calculation will include *all three* abdominal injuries.

The National Population Register was consulted to determine if the patients were alive or not at the time of the review. If the patient had emigrated, we could only ascertain that the patient was alive on date of emigration.

#### CT protocol

At the Karolinska University Hospital, the multitrauma imaging was conducted on a 256-slice multi-detector CT (MDCT) (Revolution CT, GE Healthcare, Milwaukee, Wisconsin, USA) and a 64-slice MDCT scanner (LightSpeed VCT, GE Healthcare, Milwaukee, Wisconsin, USA). During 2015 the Revolution CT scanner (RevCT) was installed and replaced the LightSpeed VCT scanner (VCT) in the trauma department. Concomitantly our single-phase (venous) standard trauma CT protocol was modified to a multi-phase protocol by including a whole-body CT in arterial phase for high-energy trauma patients. For low-energy trauma patients, a single-phase (venous) trauma CT protocol was used on the RevCT. The trauma team leader, usually the trauma surgeon on-call, determined in the trauma room if the patient should be examined according to a high-energy or low-energy trauma patient CT protocol. In the multi-phase trauma CT protocol on the RevCT, the used tube current in the arterial phase was 150 mA and in the venous phase 120 mA, and the tube voltage was 100 kV and 120 kV, respectively. For patients with obesity, the tube current in the arterial phase was 300 mA and in the venous phase 255 mA and the tube voltage was 120 kV for both phases. In the single-phase trauma CT protocol on the RevCT, the used tube current was 225 mA and the tube voltage 120 kV, and for patients with obesity 300 mA and 120 kV. In the single-phase trauma CT protocol on the VCT, the used tube current was 150–200 mA and the tube voltage 120 kV. In the multi-phase trauma CT protocol on the RevCT, the software SmartPrep^TM^ (Ge Healthcare) was used to time the arterial phase and the delay of the venous phase was 45.0 s after the end of the arterial phase. In the single-phase trauma CT protocol on the RevCT, the delay was 65.0 s, and on the VCT 55.0–60.0 s. The rotation time was 0.5 s for all protocols and enhancement phases on the RevCT, and 0.4 s on the VCT. The pitch was 0.992 for all phases on the RevCT, and 0.984 on the VCT. The detector coverage was 80 mm for all phases on the RevCT, and 40 mm on the VCT. The iodinated contrast agents ioversol (350 mg/ml) or iohexol (350 mg/ml) were used with a flow rate of approximately 4.5–5 ml/s in the CT protocol that included an arterial phase and 2.5–3.5 ml/s in the venous phase only CT protocols.

Of the entire cohort (1373 patients with a CT performed) 15 patients were assessed using thick sliced (5 mm) CT imaging. Four patients were assessed using CT without intravenous contrast, one patients imaging was a low-dose CT. When an adrenal abnormality was present, all written reports (primary and final reviews) were evaluated separately after reassessments to investigate if the adrenal glands were mentioned in the report.

#### Labelling

Adrenal findings were categorized as either adrenal tumors or hematomas. A finding in the adrenals ≥10 mm considered not to be a hematoma was labelled an adrenal lesion. Adrenal hematomas were labelled hematomas according to their size, attenuation and their shape. In certain cases, the size of the adrenal hematoma was difficult to estimate due to the trauma and current bleeding in the area. In most cases this distinction between adrenal tumor and hematoma was easily made but, in some cases, (n = 8) the differentiation was difficult. In these cases, a third radiologist (SK) re-examined the cases and a final decision was made.

#### Ethical considerations

The present study was approved by Swedish Ethical Review Authority (application number 2022-02753-02).

#### Consent to participate and publish

Due to the study’s retrospective nature informed consent was waived (application number 2022-02753-02).

#### Statistical analysis

Data are presented as mean (95% CI). Calculations were performed using a commercial software package Statistica (version 20). Frequency analysis and student t-test were performed. Histograms of the age-related distribution of adrenal tumors and hematomas and their size are presented. A p-value less than 0.05 was considered significant.

## Results

**Fig. 1:**
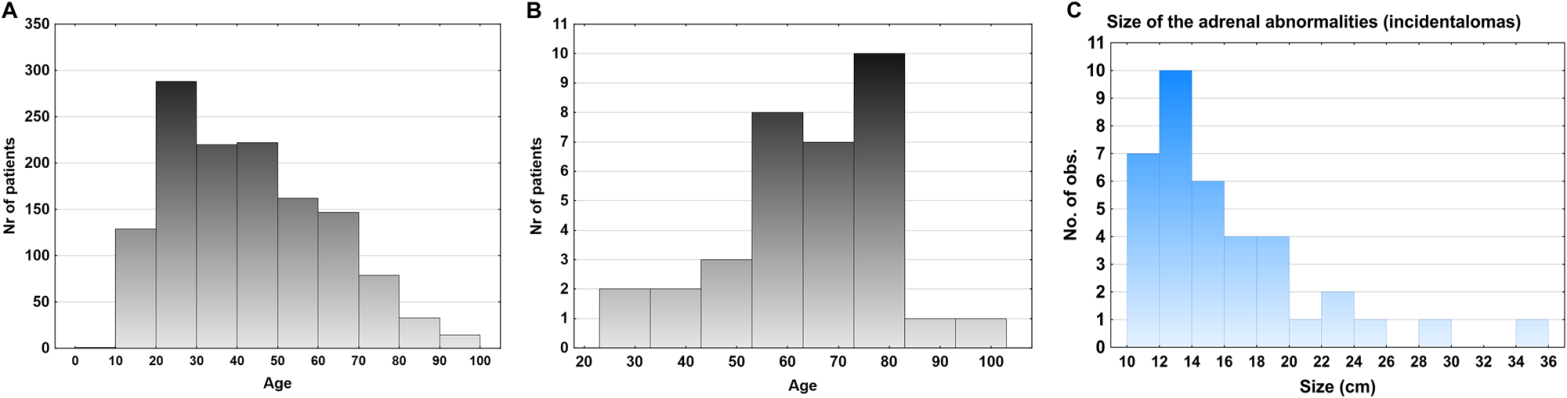
**A** The age distribution of patients with multitrauma (n = 1295). **B** The prevalence of adrenal lesions in relation to age in patients with multitrauma. An increasing number of individuals were found with older age. **C** The size of the adrenal lesions in the cohort. The majority of the adrenal lesions was less than 20 mm

In total 1373 patients with multitrauma were included, of which 37 patients (2.7%) were found to have adrenal lesions (bilateral n = 8). The distribution in age in the entire cohort are presented in Fig. 1A and the distribution of patients with adrenal lesions in relation to age and size are presented in Fig. 1B and C. The patients with adrenal lesions were older compared to those without an adrenal lesion (62 [56–68] vs 42 [41–43] years, p < 0.0001) (Table 1). There was an accumulating prevalence of adrenal lesions in relation to increasing age (Fig. 1B). There were no significant relationship or association between the adrenal lesion size and increasing age (p = 0.75).

**Table 1:** Characteristics at the time of the radiology for multitrauma patients included in the study

	Patients without adrenal abnormalities (n = 1267)	Patients with adrenal abnormality All (n = 106)	Patients with adrenal lesions (n = 37)	Patients with adrenal hematomas (n = 69)
Women (n), %)	368 (29.0)	28 (264)	12 (32)	16 (23)
Age (years)	42 (41–43)	50 (46–54)	62 (56–68)*	43 (39–47)
Weight (kg)	75 (74–76) (n = 643)	74 (70–78) (n = 56)	77 (70–84)	72 (66–77) (n = 35)
Height (cm)	176 (175–176) (n = 642)	175 (172–177)	174 (169–179)	175 (172– 178) (n = 35)
BMI	24.1 (23.8–24.5) (n = 641)	24.1 (23.0–25.2) (n = 56)	25.3 (23.8–26.9)	23.4 (21.9–24.9) (n = 35)
LOS (days)	11 (10–12)	20 (14–25)	15 (9–21)	22.3 (14.3–30.3)*
30-day mortality, n affected/total (%)	62/1260 (4.9)	8/105 (7.5)	3/37 (8.1)	5/68 (7.2) b
ISS	18 (17–19)	28 (25–32)	21 (16–26)**	33 (29–37)*
NISS	21 (20–22)	33 (29–36)	24 (18–30)***	38 (34–42)*

Data are presented as mean (95% CI) or number, (%)

*BMI* body mass index, *CI* confidence interval, *ISS* injury severity score, *LOS* length of hospital stay, *NISS* New injury severity score (calculating the patients three most severe injuries regardless of the body region in which they occur)

*p < 0.001 vs patient without adrenal abnormalities

**p < 0.01 vs adrenal hematoma group

***p = 0.001 vs adrenal hematoma group

**Fig. 2:**
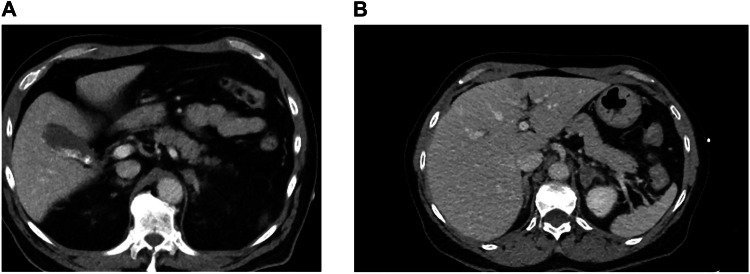
**A** CT imaging of an adrenal lesion in a patient at the initial trauma CT. A left adrenal lesion of 17 mm with an attenuation of up to 52 HU in a middle-aged woman. The left adrenal was mentioned as prominent in the initial report. No follow-up CT was performed. **B** CT performed 2 years after the trauma CT in the same patient. However, a contrast enhanced CT under another question two years later showed the lesion with similar size and a HU of 52

Of the unilateral adrenal lesions 86% (25/29) were left-sided. Of the unilateral adrenal lesions 76% (22/29) were undiagnosed and not mentioned in the report, thus only 24% was mentioned in the original imaging reports. If the size of the adrenal lesion was ≥15 mm (n = 13), 38% (5/13) was mentioned in the original imaging reports. In Fig. 2A, B CT imaging of a subject with an adrenal lesion is presented. Of the bilateral adrenal lesions 38% (3/8) had been mentioned in the original imaging reports. During the mean ( ± 95% CI) follow-up time of 75 (61–90) months none had adrenal surgery, none had apparent adrenal insufficiency nor signs of hyperfunctioning, 6% (2/36) had the adrenal hormones measured, all normal. Only 7% (2/29) had new imaging concentrating on the adrenal (Table 2). None had a review by an endocrinologist.

**Table 2:** Characteristics at the time of the radiology for multitrauma patients with adrenal lesion or hematoma

At time of radiology	Patients with adrenal lesions (n = 37)	Patients with adrenal hematomas (n = 69)	p-value
Women, n (%)	12 (32.4)	16 (23)	0.30
Size (mm)	16 (14–17)	27 (23–30) (n = 37)	<0.001
Age (years)	62 (56–68)	43 (39–47)^a^	<0.001
Flow-up time (months)	75 (61–90)	89 (80–98)^d^	0.41
New radiology, n yes/no (%)	2/27 (6,9%)^b^	22/47 (31.9%)	0.009
Surgery (% yes)	0%	0%	–
Alive, n yes/no (%)	23/13 (63.9%)^e^	60/9 (87.0%)	0.006
Adrenal failure	none	none	–
Hormones examined, n yes /no (%), all normal	2/34 (5.6%)^e^	3/57 (5.0%)^f^	0.91

Data are presented as mean (95% CI) or number, n / total (%). n number,

^a^one missing, ^b^two missing, ^c^8 missing, ^d^7 missing, ^e^one missing, one died shortly after due to trauma, ^f^9 missing, one said no to further investigation

**Fig. 3:**
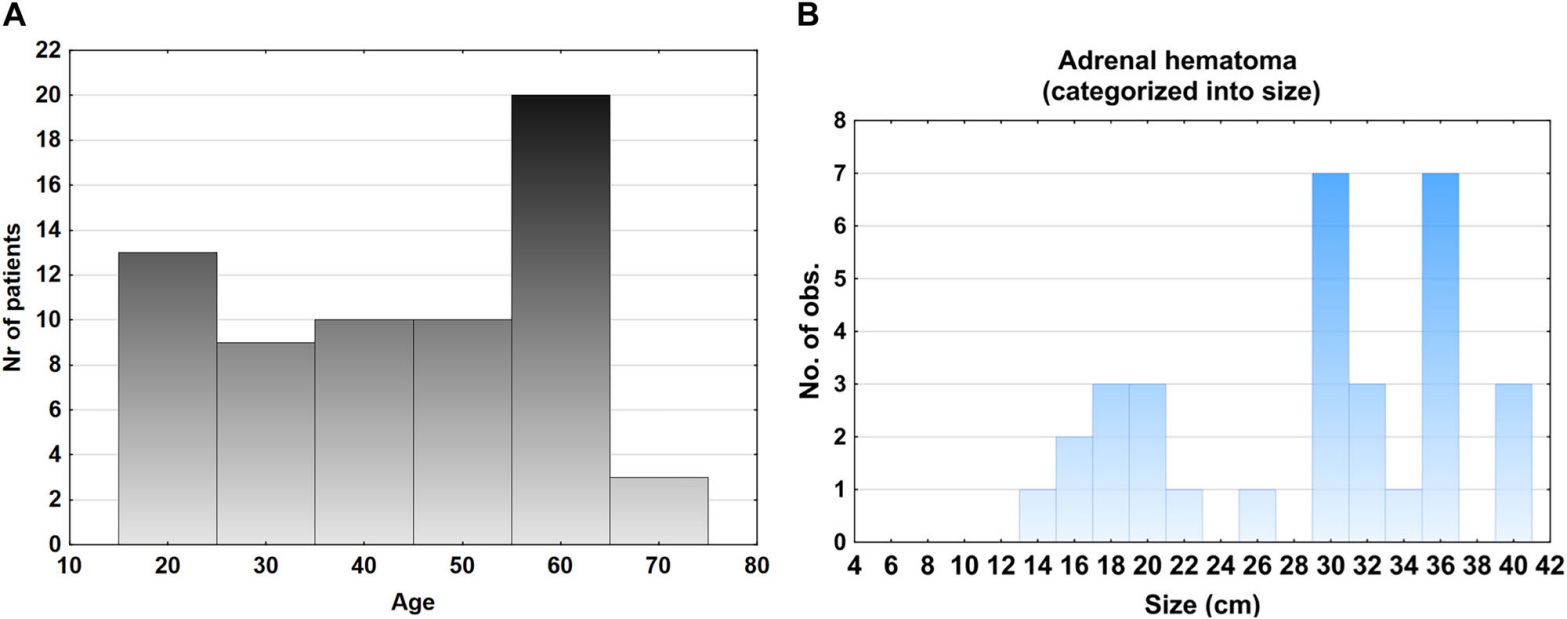
**A** The prevalence of adrenal hematoma in relation to age in patients with multitrauma. The prevalence was reflecting the age distribution in the cohort. **B** The size of the adrenal hematomas in the cohort. The majority of the adrenal hematomas in the cohort was larger than 20 mm

**Fig. 4:**
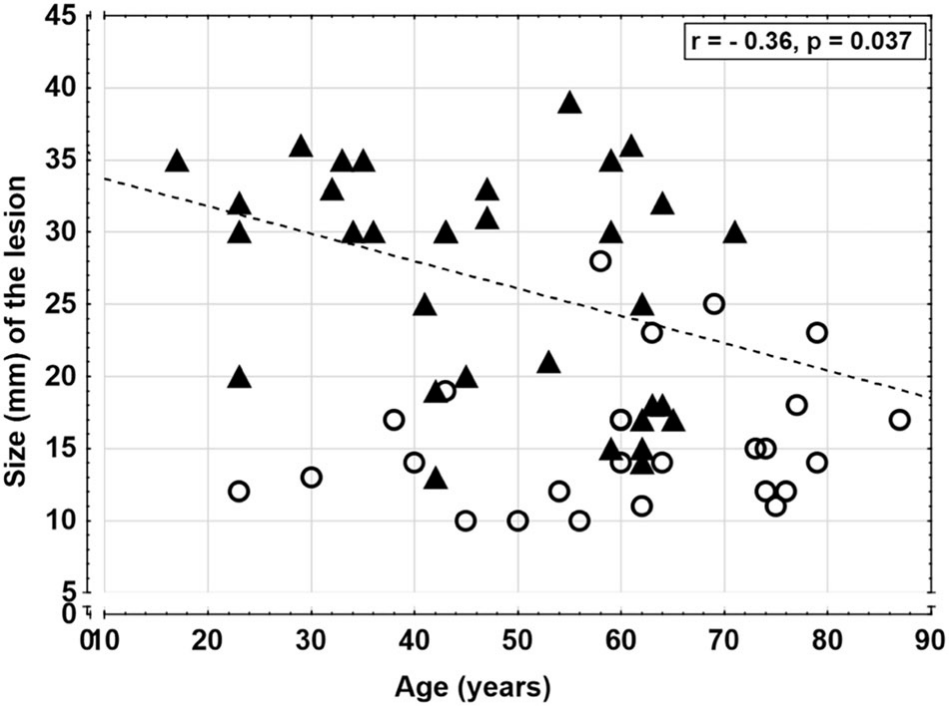
Size of the adrenal abnormalities (mm) in relation to age (years). Comparison between adrenal lesions (unfilled circles) and adrenal hematomas (filled triangles). The dotted line represents adrenal hematomas subjects **(**r= –0.36, p < 0.05), thus a significant inverse relationship between age and size of the adrenal hematomas was present in the cohort, not seen for adrenal lesions

**Fig. 5:**
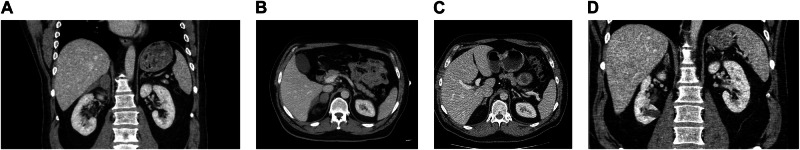
**A,** **B** CT imaging of an adrenal hematoma in a patient at the initial trauma CT. An adrenal hematoma in a middle-aged man, mentioned in the initial report. **Axial imaging:** An oval shaped lesion with an attenuation of 55 HU on contrast enhanced CT is seen in the right adrenal with a small amount of adjacent fluid (blood attenuating on CT). **C, D** Follow-up CT- imaging after 6 weeks in the same patient. **Coronar imaging**. Six weeks later a follow-up CT was performed; the adrenal lesion had significantly reduced in size, with only remaining a residual low-attenuation lesion in the right adrenal gland

We also found 69 adrenal hematomas (bilateral n = 7) in the 1373 patients (5.0%). The mean age of patients with adrenal hematomas was similar to those patients without adrenal lesions (43 [39–47] vs 42 [41–43] years, p = 0.91) (Table 1) but significantly lower compared with those patients with an adrenal lesion (p < 0.0001). There was no association between the prevalence of adrenal hematomas and age, the relationship presented in Fig. 3A, the prevalence reflecting the general age distribution. The size of the adrenal hematomas was larger than the adrenal lesions (27 [23–39] vs 16 [14–17] mm, p < 0.0001) (Table 2) and their distribution in relation to size are presented in Fig. 3B. There was a significant inverse relationship between the size of the adrenal hematomas with age (r = –0.36, p < 0.005) (Fig. 4), while this association was not seen for adrenal lesions. Of the unilateral adrenal hematomas most were right-sided (77%, 48/62). Of the unilateral adrenal hematomas 42% (26/62) was not mentioned in the initial report. Of the bilateral hematomas 57% (4/7) were not mentioned. In Fig. 5A–D CT imaging of a patient with an adrenal hematoma at initial trauma CT and at follow-up, 6 weeks later are presented, this adrenal hematoma was mentioned in the initial report. In Table 3 the cohort was also stratified by age groups <60 vs ≥60 years. The prevalence of adrenal lesions was higher in the age group ≥ 60 years (p < 0.005); and adrenal hematomas were more common in individuals less than 60 years of age (p = 0.012). The older age group had comparatively less follow-up time than the younger age group and not surprisingly fewer were alive in this group after follow-up (Table 3).

**Table 3:** Patients with adrenal abnormalities (n = 105) stratified by age groups <60 vs ≥60 years

At time of radiology	Patients <60 years (n = 66)	Patients ≥60 years (n = 39)	p-value
Women, n (%)	15 (23.4)	13 (36.1)	0.10
Size (mm)	25 (22–28)	18 (15–20)	<0.0001
Age (years)	39.6 (36–43)	69 (66–72)	<0.0001
Adrenal lesions, n (%)	13 (20)	23 (59)	0.005
Adrenal hematomas, n (%)	53 (80)	13 (33)	0.012
LOS (days)	19 (12–26)	20 (13–28)	0.90
ISS	30 (26–34)	27 (21–32)	0.34
NISS	35 (20–29)	31 (25–37)	0.22
Flow-up time (months)	91 (81–101)	73 (59–87)	0.030
New radiology, n yes/no (%)	16/36 (30.7%)^a^	7/24 (22.6%)^d^	0.29
Surgery (% yes)	0%	0%	–
Alive, n yes/no (%)	49/8 (86%)^b^	13/22 (62.8%)^e^	<0.001
Adrenal failure	none	none	–
Hormones examined, n yes /no (%), all normal	2/56 (3,2%)^c^	3/35 (8.6%)^f^	0.31

Characteristics at the time of the radiology for multitrauma patients

Data are presented as mean (95% CI) or number, n / total (%). n number

^a^14 missing ^b^11 missing, ^c^2 missing, ^d^8 missing ^e^four missing, ^f^one missing, one died shortly after due to trauma, one refused follow-up

Mortality rates in the adrenal lesion and hematoma groups were similar, but the hematoma group had higher ISS and NISS but not LOS compared with the adrenal lesion group (Table 1). After the follow-up time of 75 (61–90) months a higher proportion of patients were alive in the adrenal hematomas group (Table 2). The adrenal hematoma group had a higher incidence of follow-up radiology.

## Discussion

In this large trauma cohort, an adrenal lesion prevalence of 2.7% and an adrenal hematoma prevalence of 5.0% were found. The mean age of the entire trauma cohort was only 42 years, i.e., a relatively young cohort and the individuals were presumably otherwise healthy. Most adrenal lesions were not diagnosed in the initial radiological report but were found when we reviewed all the imaging. Very few had the adrenal hormones measured, however, none had any apparent signs of hyperfunctioning or adrenal insufficiency and this did not seem to affect short- or long-term outcomes.

Jing et al. showed a prevalence of 1.4% of adrenal lesions in a health check cohort of more than 25000 participants [6], of which approximately 70% of the adrenal lesions were non-functional. Our higher prevalence may be attributable to the fact that relatively small adrenal lesions were included. In a Swedish multicenter study all adult patients with incidentally discovered adrenal lesions detected at CT were prospectively reported from the radiology departments of all hospitals in Western Sweden (1.66 million inhabitants) for 18 months [9]. The reported frequency of adrenal lesions was 0.9% although a re-evaluation of the images of >3800 patients showed much higher percentages (1.8–7.1%). This phenomenon indicated an underestimation of the adrenal lesions that is in accordance with the result of the present study.

If we compare our findings of trauma induced adrenal hematomas found by re-evaluations with the given prevalence reported using imaging reports [10], the majority of adrenal hematomas had been missed. This may be due to that multitrauma with excessive injuries/findings in the depicted trauma CT complicates the distinction of the adrenal trauma. In a large study of 2692 trauma patients, the CT imaging was re-examined by several radiologists. They found a mean size of 28 mm [11] of the adrenal hematomas, similar as ours (27 mm) and an incidence of 1.9%. Their lower prevalence is most probably attributable to a different trauma cohort consisting of not only abdominal trauma.

Of the adrenal abnormalities found in the current study, 40% of the hematomas and 75% of the adrenal lesions were not mentioned in the radiology report, thus in many cases an adrenal abnormality was missed. For larger adrenal lesions (≧15 mm) slightly less were missed. In accordance with our results, another study of 112 patients with hypertension in which the adrenal CT were initially reported normal, a re-evaluation by multiple radiologists showed a relatively high frequency of adrenal abnormalities (10 – 50.5%) [12]. These results indicate that the adrenals might be easily missed during CT imaging and evaluation.

The adrenal hematoma group did not differ in age compared with the rest of the cohort. Not surprisingly, patients with adrenal lesions had a higher average age. Patients with multitrauma and adrenal hematomas had longer hospital stay, and higher injury severity score compared with patients with multitrauma and adrenal lesion and compared with patients with multitrauma and no adrenal lesions. Survival rates between the adrenal lesion and adrenal hematoma groups did not differ. Thus, those patients with multitrauma and adrenal hematoma seemed to have been exposed to higher trauma impact, but it did not seem to affect survival.

Of the unilateral adrenal lesions 86% were left-sided whereas 77% of the adrenal hematomas were right sided. Adrenal lesions are more common on the left side which might be attributed to limited space on the right side because of the liver. This has been seen in other studies in which adrenal lesions are more likely to be identified on the left adrenal than the right [4, 13]. Hematomas are more often right-sided probably because the right adrenal gland becomes more compressed between ribs and liver compared to the left adrenal gland in a trauma to the area. A higher incidence of right-sided hematomas was found in two other studies, with similar figures as ours (82% and 76.6% of the adrenal hematomas, respectively, were right-sided) [11, 14]. Even in a study of adrenal hematoma in which trauma cases accounted for 47% of the cohort, right-sided hematomas were more common [7].

Few patients in the adrenal lesion group, less than 10%, had follow-up radiology, and only 6% had hormones measured. Adrenal lesions are most often silent adrenal masses and only in a few cases do overt hyperfunction develops [2, 4, 15]. Not surprisingly the adrenal hematoma group had a higher incidence of follow-up radiology compared with the adrenal lesion group. The adrenal lesion and hematoma groups did not differ in 30-day survival rates. Differences in survival likely reflect confounding factors such as age and trauma severity, not adrenal pathology.

The main aims when encountering an adrenal lesion are to rule out malignancy [1]. Adrenocortical cancer and particularly adrenal metastases have high mortality rates [4, 16]. Functional adrenal tumors, particularly pheochromocytomas can lead to serious complications and even death if left undiagnosed [17]. Thus, evaluating an adrenal lesion is of utmost importance even though the majority are nonfunctional adrenal adenomas where it may not matter that much if the adrenal tumor is missed. It should be noted that bilateral adrenal metastases or adrenal hematomas may result in adrenal insufficiency [18, 19]. In view of our low follow-up figures, 75% of adrenal lesions were not evaluated further with hormonal assessment, and hormonal assessments it is recommended that an endocrinologist should be consulted every time an adrenal pathology is found. This is line with recommendations from the ESE guidelines [1]. A close collaboration between trauma radiologists and endocrinologists is likely to be important in improving management. None of our patients with adrenal lesion seemed to have suffered because of the missed diagnosis, however, since few had hormones measured and follow-up was limited some may have suffered without our knowledge.

### Limitations

Like all studies the present study had some limitation. Similar to other retrospective studies we had to rely on existing medical records and missing data were an issue. The study was dependent on the accuracy and completeness of the medical records. All CTs except for four patients in the cohort were performed with intravenous contrast making it difficult to establish the exact HU of the adrenal abnormality. In 15 cases thin imaging CT were lacking, and thick slices (2–5 mm) were used to assess the adrenals. This might have affected our results. In one of these 15 patients an adrenal lesion was present. One experienced radiologist examined all patients, and another re-examined all patients in whom an abnormality was found. All radiology reports where an abnormal adrenal finding was present were re-examined. The limited hormonal work-up (6% of cases) and complete absence of endocrine referral significantly weakens the ability to draw conclusions about the clinical impact.

## Conclusion

In this large cohort of multitrauma patients the prevalence of adrenal lesions were 2.7% and adrenal hematomas 5.0%. Most adrenal abnormalities were not diagnosed during the admission and were found only when we re-examined all the abdominal imaging. Patients with adrenal lesions were older while patients with adrenal hematoma had similar age as the patients without adrenal abnormalities. Adrenal hormones were rarely measured, and no patient was reviewed by an endocrinologist which makes is difficult to predict the clinical significance of adrenal lesions in a trauma setting.

## Data Availability

Data can be available upon request to the corresponding author.

## Abbreviations

CT: Computed tomography
ISS: injury severity scale
LOS: Length of hospital stay
MASCS: mild autonomous cortisol secretion
NISS: New injury severity scale
PACS: Picture archiving and communication system
RIS: Radiological information system
