# Correction: Adrenal lesions in patients with abdominal multitrauma

**DOI:** 10.1007/s12020-025-04443-0

**Published:** 2025-10-03

**Authors:** Anna Kistner, Lisa Kekonius, Jan Calissendorff, Seppo Koskinen, Henrik Falhammar

**Affiliations:** 1 Department of Molecular Medicine and Surgery, Karolinska Institute, Stockholm, Sweden; 2 Department of Nuclear Medicine and Medical Physics, Karolinska University Hospital, Stockholm, Sweden; 3 Department of Endocrinology, Karolinska University Hospital, Stockholm, Sweden; 4 Department of Clinical Science, Intervention, and Technology, Division of Radiology, Karolinska Institute, Stockholm, Sweden; 5 Department of Radiology, Karolinska University Hospital, Stockholm, Sweden

Correction to: Endocrine (2025) 90:321–328

10.1007/s12020-025-04321-9

In this article the author’s name “Jan Calissendorff” was incorrectly written as “Jan Calissendorf”.

The original article has been corrected.

